# A perforation of a duodenal diverticulum in a 97-year-old patient after total gastrectomy and Roux-en-Y reconstruction: a case report

**DOI:** 10.1007/s12328-024-01965-4

**Published:** 2024-04-08

**Authors:** Shinya Ohno, Tomohito Shinoda, Tatsuki Kawahara, Yusuke Nonomura, Reo Tachikawa, Kakeru Tawada, Aiko Ikawa, Bun Sano

**Affiliations:** Department of Surgery, Takayama Red Cross Hospital, 3-11 Tenmanmachi, Takayama, Gifu, 506-0025 Japan

**Keywords:** Diverticulectomy, Duodenal closure, Super-elderly patient, Duodenal stump

## Abstract

Most duodenal diverticula (DD) are asymptomatic and rarely develop perforations. Perforation is the most serious complication of DD and often requires emergency surgery. A 97-year-old woman who had undergone total gastrectomy and Roux-en-Y reconstruction 30 years ago was referred to our department with chief complaints of abdominal pain and fever during her hospitalization after femoral neck fracture surgery in the orthopedic department. Contrast-enhanced computed tomography showed free air and residue in the abdominal cavity and right retroperitoneum, and an emergency laparotomy was performed. The abdominal cavity was mildly contaminated, and a 6-cm DD with a 1-cm perforation in the wall of the diverticulum on the contralateral side of the mesentery of the duodenum was found. Diverticulectomy and duodenal closure were performed and a drainage tube was placed. The patient experienced no complications and was transferred to the orthopedic department on postoperative day 10. Reports of perforation of DD after gastrectomy are very rare. Particular attention should be paid to perforation of DD after Billroth-II and Roux-en-Y reconstructions as they involve the formation of a duodenal stump that differs from the normal anatomy and may be highly invasive surgical procedures, depending on the degree of inflammation and fistula formation.

## Introduction

A duodenal diverticulum (DD) is the second most common diverticulum occurring in the gastrointestinal tract after colonic diverticulum, with a prevalence of 1–2% on upper gastrointestinal series and 4–22% at autopsy [1, 2]. The most frequent location is the second portion of the duodenum. Around 95% of the patients are asymptomatic, and complications are reported in 5–10% of patients [3]. Major complications include biliary or pancreatic duct obstruction, hemorrhage, diverticulitis, Lemmel’s syndrome and perforation [4–6]. Perforation of a DD is the most serious complication, with diverticulitis being the most common cause, followed by enterolithiasis, iatrogenic origin, ulceration, traumatic injury, and foreign body [7]. Right upper quadrant pain with nausea and vomiting is one of the most frequent symptoms. Because a DD may cause abdominal pain with signs of peritoneal irritation but may only cause perforation to the retroperitoneum and not lead to panperitonitis, the symptoms of DD are frequently nonspecific, often making preoperative diagnosis difficult [8–10].

Reports of perforation of DD after gastrectomy are very rare. Particular attention should be paid to DD perforation after Billroth-II and Roux-en-Y (R-Y) reconstructions as they involve the formation of a duodenal stump that differs from the normal anatomy and may be highly invasive surgical procedures, depending on the degree of inflammation and fistula formation.

We report a very rare case of perforation of a DD after R-Y reconstruction in a super-elderly patient who was successfully treated surgically.

## Case report

A 97-year-old woman who had undergone orthopedic surgery for a femoral neck fracture with open osteosynthesis and gamma nailing was referred to our department with chief complaints of abdominal pain and fever. Her medical history included a total gastrectomy 30 years ago with unknown details and chronic atrial fibrillation. The patient was taking an anticoagulant, non-steroidal anti-inflammatory drug, and proton pump inhibitor.

**Fig. 1:**
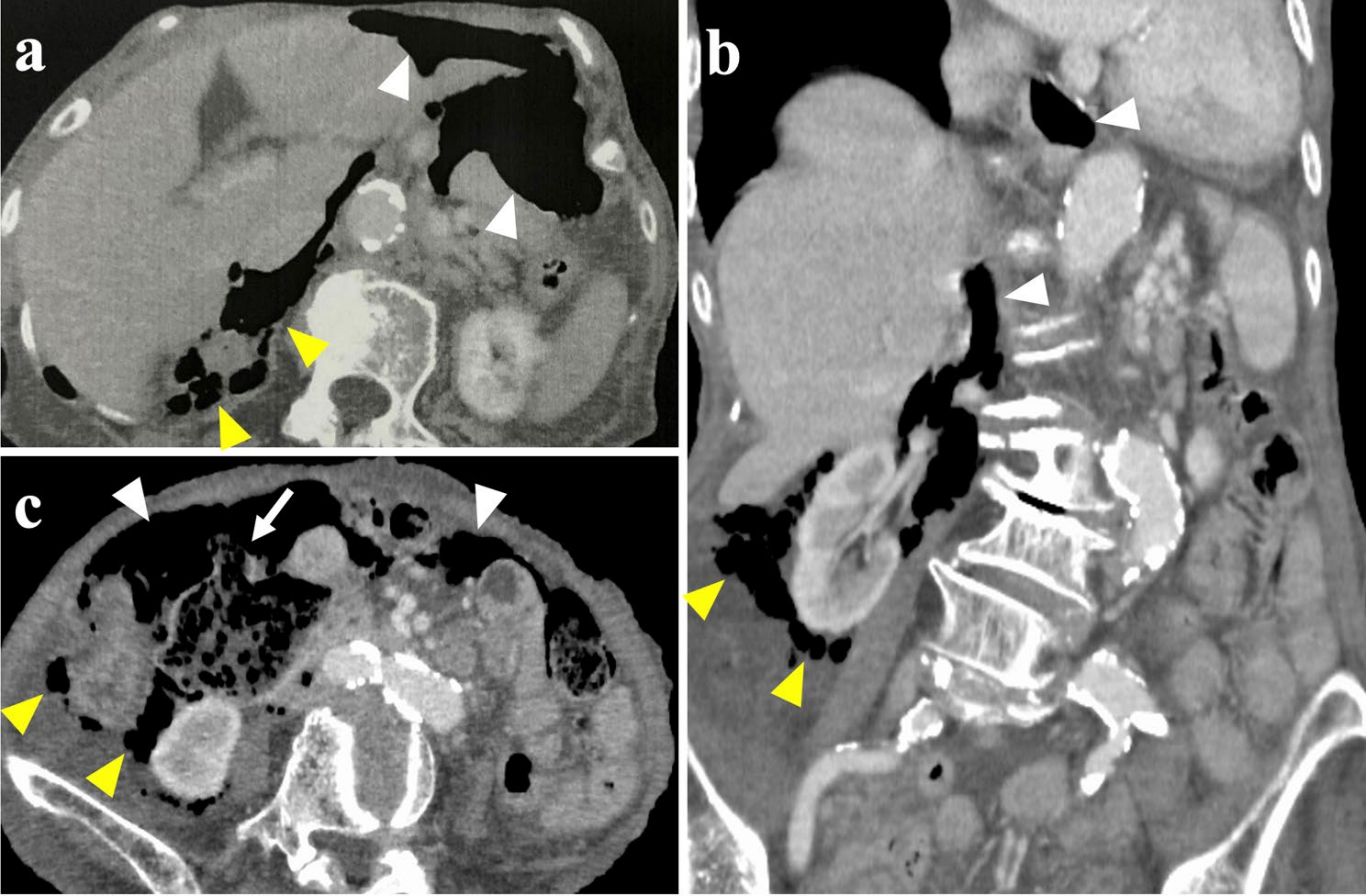
Contrast-enhanced computed tomography (CT) at patient referral to our department. **a, b** The axial and coronal CT images revealed air in the abdominal cavity (white arrowheads) and right retroperitoneum (yellow arrowheads) and **c** residue into the abdominal cavity (white arrow) (color figure online)

**Fig. 2:**
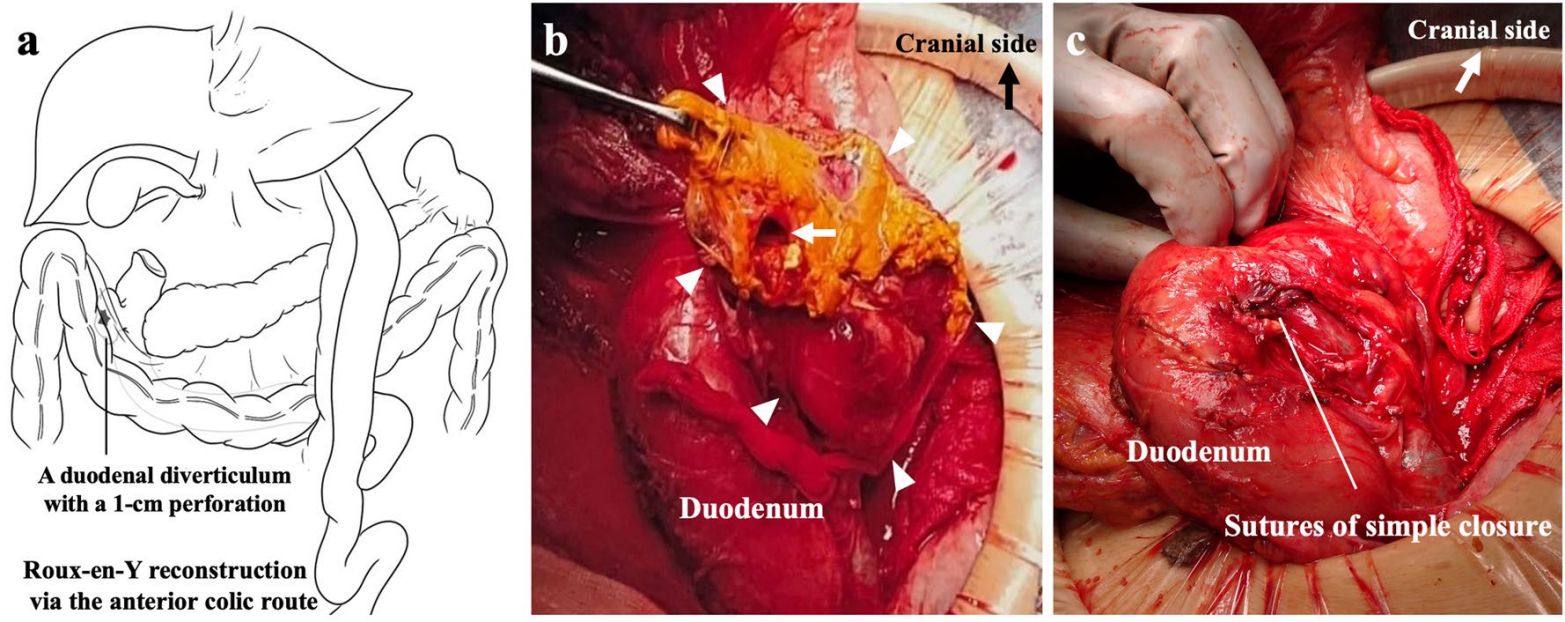
Surgical illustrations and intraoperative findings. **a** Illustration of the DD after the previous total gastrectomy and Roux-en-Y reconstruction. **b** A 6 cm duodenal diverticulum (DD) (arrowheads) and 1 cm perforation in the wall of the diverticulum on the contralateral side of the mesentery of the duodenum (arrow) were found. **c** After the diverticulectomy and simple duodenal closure. Sutures were on the contralateral side of the mesentery of the duodenum

**Fig. 3:**
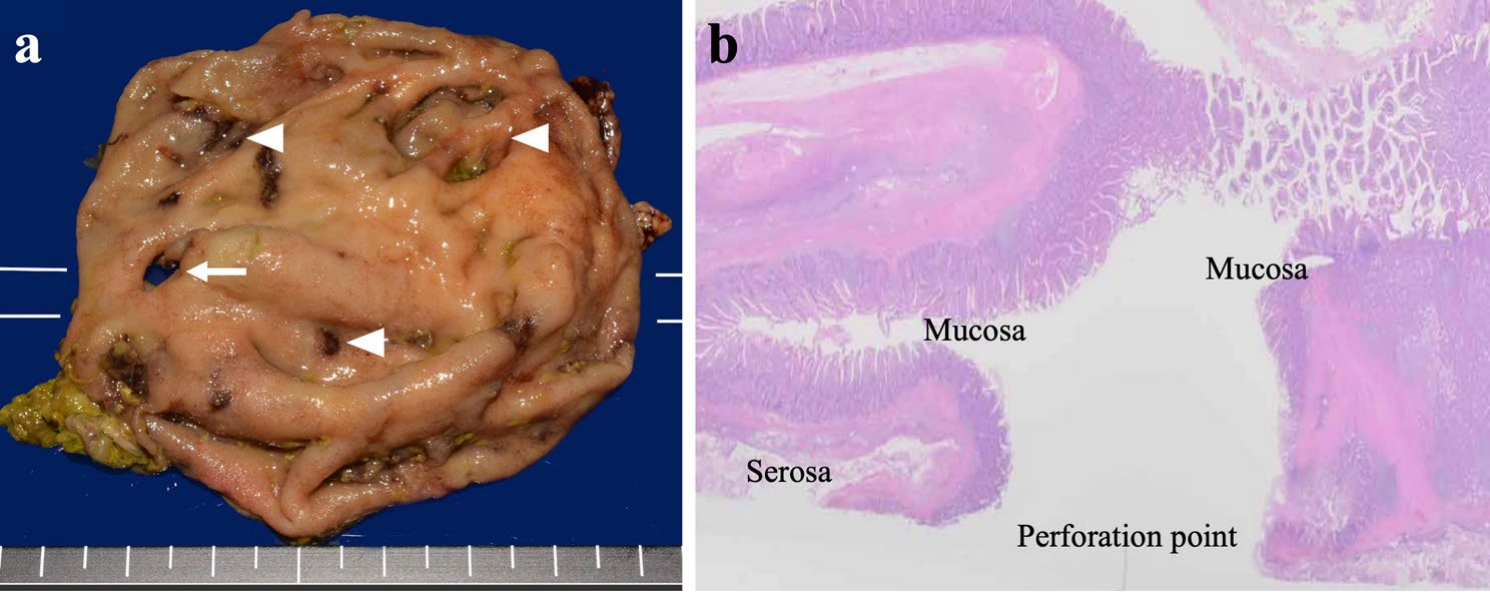
Histopathological findings. **a** Partially thinned regions were observed in the true diverticulum (arrowheads), although the laminar structure was preserved. A 1-cm perforation was present in the wall of the diverticulum (arrow). **b** The perforated region showed rupture of all layers

The patient developed sudden abdominal pain 9 days after orthopedic surgery and was referred to our department 3 h after the onset of abdominal pain. At referral to our department, the patient was 139 cm tall, weighed 32.2 kg, body mass index was 16.6 kg/m^2^, temperature was 38.6 ºC, pulse was 68 bpm, and blood pressure was 120/48 mmHg. Right lower abdominal pain was noted, but there were no signs of perineal irritation. Slightly elevated markers of inflammation and anemia were apparent, with a white blood cell count of 4740/μL, a C-reactive protein value of 2.12 mg/dL and hemoglobin of 9.0 g/dL; liver enzymes and creatinine were within normal limits. Contrast-enhanced computed tomography showed free air and residue in the abdominal cavity and right retroperitoneum (Fig. 1a-c), but it was difficult to identify the site of perforation, and an emergency laparotomy was performed for suspected DD perforation or the lower gastrointestinal tract perforation. At surgery, her abdominal cavity was mildly contaminated, a 6-cm DD located 30 cm from Y-anastomosis, and the DD with a 1-cm perforation in the wall of the diverticulum on the contralateral side of the mesentery of the duodenum were found, with the interior of the DD filled with food residue (Fig. 2a,b). We performed the diverticulectomy and duodenal closure (Fig. 2c) with placement of an infrahepatic drainage tube from the right side of the abdomen. Histopathological findings of the DD showed a true diverticulum with partial wall thinning and rupture (Fig. 3), suggesting perforation of the diverticulum due to increased internal pressure and chronic inflammation caused by food residue for many years. Oral intake was started on postoperative day (POD) 3. The drainage tube was removed on POD 5. The patient experienced no complications and was transferred to the orthopedic department on POD 10.

## Discussion

Reconstruction procedures for gastric cancer include the Billroth-I, Billroth-II, and R-Y reconstruction, with Billroth-II and R-Y reconstruction having an afferent loop and duodenal stump. Postoperative complications of R-Y reconstruction include Roux stasis syndrome, Petersen’s hernia, and duodenal stump leakage [11], with the latter considered to be caused by increased pressure at the edge of the stump due to intestinal peristalsis [12, 13]. To our knowledge, there have been only two reports of perforation of DD after gastric cancer surgery, one case with Billroth-II reconstruction [14] and two cases with R-Y reconstruction [15], both of which were considered to be caused by increased pressure in the afferent loop of the duodenum. Our patient was receiving postoperative care following repair of a femoral neck fracture, and there was food residue in the DD. In addition to weakening of the diverticular wall due to chronic inflammation caused by food residue remaining in the DD for many years, the temporary decrease in intestinal peristalsis caused by the physical stress of femoral bone surgery, especially the decrease in duodenal peristalsis, was considered to have increased pressure in the duodenum and caused the perforation of the DD.

Treatment options for perforation of DD can be conservative or surgical. Conservative treatment may be chosen for patients with micro-perforation, localized inflammation or abscess, and stable general condition with few symptoms [3, 16], but surgical treatment should be chosen for patients with panperitonitis or sepsis or those with significant leakage of intestinal contents due to perforation who are not expected to improve with conservative treatment. The first surgical treatment for perforation of DD is diverticulectomy and two-tiered duodenal closure [17–19]. However, in cases of severe inflammation of the duodenum and insecure closure or weakness of the duodenal wall, a duodenojejunostomy may be needed to reduce the risk of duodenal leakage or fistula. Although variations from R-Y duodenojejunostomy with gastro-jejunal exclusion to truncal vagotomy/antrectomy with Billroth II gastrojejunostomy or even pancreaticoduodenectomy have been reported [15, 20–22], an increase in surgical complications due to the high invasiveness of the procedure is a concern. Intra-abdominal drainage and feeding jejunostomy have also been reported to improve symptoms [23], and surgery such as surgical drainage or tube-duodenostomy is an option for older patients who are not expected to improve with conservative treatment and cannot tolerate highly invasive surgery or whose general condition is unstable. Fortunately, although our patient was 97 years old, she was still able to tolerate the surgery, which was completed with only a diverticulectomy and two-tiered duodenal closure. Because she had previously undergone total gastrectomy and R-Y reconstruction, gastrojejunostomy was not feasible. In addition, due to her age, it was difficult to perform highly invasive surgery such as pancreatoduodenectomy. If simple closure is not feasible because of excessive inflammation and a large DD fistula, palliative surgery such as surgical drainage or duodenojejunostomy, or biliary drainage through the biliary duct with cholecystectomy, may be considered.

In conclusion, in patients with total gastrectomy and R-Y reconstruction who develop a DD, the possibility of perforation due to increased intraduodenal pressure should be considered.

## Abbreviations

DD: Duodenal diverticulum
R-Y: Roux-en-Y
POD: Postoperative day
